# A Rare Case of Multiple Myeloma Presenting as Evan’s Syndrome

**DOI:** 10.1177/2324709619852760

**Published:** 2019-06-14

**Authors:** Anthony Karapetians, Tushar Bajaj, Amanda Valdes, Arash Heidari

**Affiliations:** 1 UCLA—Kern Medical, Bakersfield, CA, USA; 2 Ross University, Miramar, FL, USA

**Keywords:** multiple myeloma, Evans syndrome, anemia, thrombocytopenia

## Abstract

Multiple myeloma is defined as the neoplastic proliferation of plasma cells resulting in a monoclonal gammopathy. The classic presentation of a patient is someone who presents with bone pain, osteopenia, or new onset fractures. We present a case of multiple myeloma presenting as Evan’s syndrome (ES). Evan’s syndrome is autoimmune hemolytic anaemia with autoimmune thrombocytopenia. A 44-year-old female was referred from her primary physician to the hospital as laboratory testing revealed haemoglobin of 5 gm/dL. The patient reported a two-month history of fatigue and a sixty-pound weight loss. Laboratory results demonstrated autoimmune hemolytic anaemia, C3 positivity, elevated immunoglobulin (Ig)G, elevated lactate dehydrogenase (LDH), low haptoglobin, elevated reticulocyte count, elevated RDW-CV (red blood cell distribution width-corpuscular volume), positive direct Coombs test, thrombocytopenia, and proteinuria, all of which led to an underlying ES. The patient was started on intravenous steroids followed by oral steroids. A flow cytometry, serum protein electrophoresis, and cytogenetics were obtained. A bone marrow biopsy revealed multiple myeloma and she was started on Bortezomib treatment. We present the fifth reported case of Evan’s syndrome and multiple myeloma.

## Introduction

Multiple myeloma (MM) is defined as the neoplastic proliferation of plasma cells resulting in a monoclonal gammopathy. The classic presentation is a patient who presents with bone pain and associated osteolytic lesions, osteopenia, or fractures in addition to a monoclonal protein found in the serum or urine. In this article, we present a case of MM that presented as Evan’s syndrome (ES): autoimmune hemolytic anemia (AIHA) with autoimmune thrombocytopenia.

## Case Presentation

**Figure 1.**
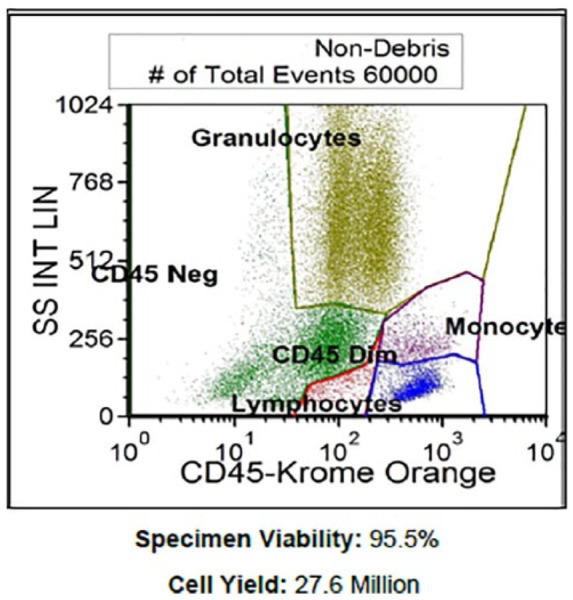
Flow cytometry.

A 44-year-old female went to her primary care physician for a regular visit and subsequently laboratory work obtained post visit revealed a hemoglobin of 5 g/dL. The patient was called immediately and urged to visit the nearest emergency department. The patient endorsed a 2-month history of fatigue and unintentional 60 pounds weight loss. Laboratory results demonstrated AIHA, C3 positivity, elevated immunoglobulin (Ig)G, elevated lactate dehydrogenase (LDH), low haptoglobin, elevated reticulocyte count, elevated RDW-CV (red blood cell distribution width-corpuscular volume), positive direct Coombs test, thrombocytopenia, and proteinuria, all of which led to an underlying ES. The patient was started on high-dose methylprednisolone 500 mg intravenous for 2 days, followed by oral prednisone taper; computed tomography chest/abdomen/pelvis with contrast was obtained for new band-like pain wrapping around the chest, which revealed a compression fracture of the L1 vertebral body. A bone marrow biopsy of the left posterior superior iliac spine was obtained demonstrating plasma cell myeloma making up greater than 80% of the marrow elements in areas with other areas less involved by the plasma cells. Additionally, the bone marrow biopsy revealed absent iron deposits in the marrow as well as normal myeloid, erythroid, and megakaryocytic elements. Flow cytometry was performed demonstrating monoclonal plasma cells, which comprised 20.3% of the total cells (Figure 1). Plasma cells showed cytoplasmic kappa light chain restriction and showed CD19 neg, CD20 neg, CD38 bri, CD45 dim to neg, CD56 neg, CD138 mod, and cKappa mod (Table 1). The serum protein electrophoresis confirmed monoclonal gammopathy with IgG-K. Quantitative IgG at presentation was 7468 with very low IgA and IgM, markedly elevated Kappa free light chain of 1879, and elevated β-2-microglobulin of 6.99. Cytogenetics were normal. As her hospital course continued, her reticulocyte count normalized following multiple blood transfusions, and her pain gradually resolved. She was initiated on bortezomib treatment prior to discharge with close outpatient hematology-oncology follow-up.

**Table 1.** Markers Performed in Flow Cytometry.

Markers Performed: CD2, CD3, CD4, CD5, CD7, CD8, CD10, CD11c, CD13, CD14, CD16, CD19, CD20, CD23, CD33, CD34, CD38, CD45, CD56, CD64, CD117, CD138, cKappa, cLambda, HLA-DR, Kappa, and Lambda (27 Markers)						
*Lymphocytes*						
CD2	CD3	CD4	CD4+/CD8+	CD5	CD7	CD8
90%	78%	34%	0.9	73%	73%	39%
CD10	CD11c	CD16	CD19	CD20	CD23	CD38
0%	13%	9%	3%	3%	1%	30%
CD45	CD56	CD56+CD3-	Kappa	Lambda	Kappa/Lambda	
100%	22%	12	2%	1%	1.4	
*Monocytes*						
CD2	CD3	CD4	CD10	CD11c	CD13	CD14
0%	1%	66%	1%	90%	90%	87%
CD16	CD19	CD33	CD34	CD38	CD45	CD56
4%	1%	91%	0%	93%	100%	2%
CD64	CD117	HLA-DR				
92%	1%	72%				
*Granulocytes*						
CD10	CD11c	CD13	CD14	CD16	CD19	CD33
12%	43%	49%	1%	63%	0%	89%
CD34	CD45	CD56	CD64	CD117	HLA-DR	
0%	100%	4%	79%	1%	1%	
*CD45 dim*						
CD2	CD3	CD5	CD7	CD10	CD13	CD14
3%	1%	1%	2%	2%	84%	3%
CD16	CD19	CD19+/CD10+	CD20	CD33	CD34	CD38
1%	3%	0	1%	9%	12%	96%
CD45	CD56	CD64	CD117	HLA-DR		
100%	3%	27%	10%	18%		
*Plasma cells*						
CD19	CD20	CD38	CD45	CD56	CD117	CD138
4%	0%	100%	94%	0%	3%	78%
cKappa	cLambda	cKappa/cLambda				
98%	0%	425.0				

## Discussion

Evans syndrome is an aberrant autoimmune disorder characterized by the combination of 2 serious hematological disorders known as AIHA and immune thrombocytopenic purpura (ITP). In 1951, Robert Evans defined this disease when studying both AIHA and ITP, dividing 5 groups of patients and verifying the presence of a serum agglutinating factor in patients with ITP.^1^ The diagnostic criteria used to depict ES consisted of elevated reticulocytes, anemia, antibodies against erythrocytes, purpura, increased blood bilirubin and fecal urobilinogen, and prolonged bleeding time. Risk factors considered in the etiology of ES include autoimmune conditions, malignancies, infections, certain medications, or recent vaccines. ES is a chronic disorder with frequent remissions and exacerbations. It is typically characterized as primary when there is no other etiology at play, or secondary when combined with other autoimmune hematologic conditions.^2^ Although frequency is unknown, research shows a higher rate in female patients.^1^ MM is a rare form of malignancy; it is otherwise considered the most common hematologic malignancy in which plasma cells undergo a characteristic monoclonal proliferation within the bone marrow. Typical manifestations of MM include hypercalcemia, renal insufficiency, anemia, lytic bone lesions, and the required diagnostic evidence of end-organ damage. It is currently unclear as to whether there is a distinct association between AIHA and MM. The risk factors responsible for ES discussed above correlate with one particularly significant risk factor of our patient had: malignancy, as with the diagnosis of MM.

Multiple myeloma is a malignancy primarily of B-cells, an abnormal process that involves the monoclonal proliferation of plasma cells. Furthermore, patients diagnosed with MM often have a precursor known as monoclonal gammopathy of undetermined significance (MGUS). Our particular patient presented with symptoms commonly seen in AIHA and ITP, prompting us to delve further into the laboratory findings. Our patient was anemic, thrombocytopenic, and exhibited severe generalize hip bone pain. The diagnosis of MM with AIHA and ITP was made in our patient without the MGUS diagnosis, diverting us to the diagnosis of ES. Although there are several laboratory findings and clinical manifestation that contribute to establishing the diagnosis of ES, the actual pathophysiologic mechanism remains unclear. Several current molecular theories in children have been studied, suggesting mechanisms that involve gain of function mutations in CTLA-4, LRBA, KRAS, and STAT3. Deficiency in TPP 2, an essential molecule that allows for cell survival during times of stress, is associated with increased levels of autoantibodies, leading to an increased risk for infections of viral origin and the development of autoimmunity. Another possible mechanism contributing to the pathophysiology of ES involves overproduction of cytotoxic T cells as well as a decreased ratio of CD4/CD8 cells, demonstrating that there are immune autoregulatory abnormalities in ES patients with low CD4/CD8 and increased numbers of T cytotoxic lymphocytes.

As ES encompasses both AIHA and ITP, we can therefore combine the diagnostic criteria for both to derive a diagnosis of ES. It is also important to note that ES is often a diagnosis of exclusion, and secondary causes of ES should be considered due to the variability in treatment and responses between primary and secondary ES. Diagnostic features of ES include a complete blood count (CBC) and direct visualization of peripheral blood.^1^ In order to arrive at a diagnosis of ES, the most useful initial laboratory tests include a CBC, reticulocyte count, blood smear, blood group and cell antigen typing, total and indirect bilirubin, LDH, haptoglobin, and direct or indirect antiglobulin test. A low hemoglobin and platelet count, elevated reticulocytes, increased indirect bilirubin, increased LDH, positive direct antiglobulin test, polychromasia, and spherocytes detected on peripheral blood smear are the resultant findings that would point us in the direction of establishing a diagnosis of ES. Our patient initially presented with severe anemia, thrombocytopenia, positive Coombs test, and elevated reticulocyte count, and she was started on high-dose steroids. Improvements in our patient’s laboratory values after obtaining these results allowed us to then investigate further as to whether this is primary or secondary ES. Differentiating between primary and secondary ES involves ruling out other conditions, such as autoimmune diseases, immunodeficiency, *Mycobacterium tuberculosis*, hepatitis C, cytomegalovirus, chicken pox, or certain malignancies that include MM, lymphoma, and giant cell hepatitis. In our patient, the diagnosis of MM was made, contributing to a possible secondary cause of ES. Although our patient met all the pertinent diagnostic criteria for ES and MM, several studies could have been obtained to further solidify our diagnosis, including the gain of function mutations discussed above that play a role in the development of ES. Furthermore, as our patient presented with clinical and categorical features of AIHA and ITP, determining TTP 2 levels in our patient could have provided further evidence of our patient’s development of autoimmunity.

Our patient initially presented with symptoms contributing to the diagnosis of AIHA and ITP: symptoms of fatigue and reported 60-lb weight loss, laboratory evidence of severe anemia with significantly decreased hemoglobin level, elevated retic count, thrombocytopenia, and positive Coombs test. In addition, following the diagnosis, our patient presented with new onset of band-like pain at the L1 level, was worked up for malignancy, and found to have IgG predominant MM diagnosed via urine and serum electrophoresis, and confirmed with bone marrow biopsy. Thus, the diagnosis of ES was made, as our patient had both autoimmune findings consistent with AIHA and ITP, and the malignant findings consistent with MM. It is worth noting that secondary causes of ES were taken into account, and in our patient with lumbar tenderness, the diagnosis of MM is a working differential in a possible secondary cause of ES. First-line treatment for ES includes high-dose steroids, which is what our patient was treated with initially. Many patients, however, have a poor response to treatment, and are often refractory, as this regimen is used to treat isolated AIHA. Our patient responded well initially to corticosteroids, allowing for the continuation of this regimen in treating ES. When she presented with bone pain symptoms, and the diagnosis of MM was established, the treatment was altered to reflect the new diagnosis.

## Conclusion

There have only been 4 other case reports of ES and MM presenting in a patient.^3-6^ We present the fifth case in a patient with a classic presentation of a rare disease phenomenon. It is important to evaluate the patient after a thorough history and physical examination with a CBC, peripheral blood smear, reticulocyte count, blood group and cell antigen typing, total and indirect bilirubin, lactate dehydrogenase, haptoglobin, and direct or indirect antiglobulin test in order to establish ES. Furthermore, there should be an investigation of the underlying cause to establish between primary versus secondary causes. In our case, we present a patient eventually diagnosed as MM with secondary ES. Treatment options for ES is with high-dose steroids; however, if the cause is secondary, the primary disease process should be treated.
